# The prevalence of foot pain and association with baseline characteristics in people participating in education and supervised exercise for knee or hip osteoarthritis: a cross-sectional study of 26,003 participants from the GLA:D® registry

**DOI:** 10.1186/s13047-023-00673-5

**Published:** 2023-11-23

**Authors:** Lucy S. Gates, Lindsey Cherry, Dorte T. Grønne, Ewa M. Roos, Søren T. Skou

**Affiliations:** 1 MRC Lifecourse Epidemiology Centre, University of Southampton, Southampton, UK; 2 School of Health Sciences, University of Southampton, University Road, Southampton, SO17 1BJ UK; 3 Solent NHS Trust, Southampton, UK; 4 Research Unit for Musculoskeletal Function and Physiotherapy, Department of Sports Science and Clinical Biomechanics, University of Southern Denmark, Odense, Denmark; 5 The Research and Implementation Unit PROgrez, Department of Physiotherapy and Occupational Therapy, Næstved-Slagelse-Ringsted Hospitals, Næstved, Denmark

**Keywords:** Osteoarthritis, Knee, Hip, Foot, Pain, Cross-sectional

## Abstract

**Background:**

Osteoarthritis (OA) affecting the knee or hip is highly prevalent in the general population and has associated high disease burden. Early identification of modifiable risk factors that prevent, limit, or resolve disease symptoms is critical. Foot pain may represent a potentially modifiable factor however little is known about the prevalence of foot pain in people with knee or hip OA nor whether foot pain is associated with clinical characteristics. The main aim of this study was therefore to determine the prevalence of foot pain in people with knee or hip OA attending an education and supervised exercise-based intervention in Denmark (GLA:D®) and determine if baseline demographic or clinical characteristics are associated with foot pain.

**Methods:**

Analysis was conducted on baseline data of 26,003 people with symptomatic knee or hip OA completing a pain mannequin as part of the Good Life with osteoArthritis in Denmark (GLA:D®) primary care programme. Odds Ratios (OR) and 95% confidence intervals (CI) were calculated to estimate the strength of association between baseline clinical characteristics (including pain severity in worst knee/hip joint, number of painful knee/hip joints, pain medication use and physical activity level) and the presence of baseline foot pain.

**Results:**

Twelve percent of participants (*n* = 3,049) reported foot pain. In those people with index knee OA (*n* = 19,391), knee pain severity (OR 1.01 CI 1.00, 1.01), number of painful knee/hip joints (OR 1.67 CI 1.58, 1.79), and use of pain medication (OR 1.23 CI 1.12, 1.36) were statistically associated with foot pain. Excluding use of pain medication, similar associations were seen in those with index hip OA.

**Conclusion:**

Twelve percent of people with knee or hip OA participating in GLA:D® had foot pain. Those with worse knee/hip pain, and greater number of painful joints were more likely to report foot pain. This study is the first to demonstrate a significant relationship between clinical characteristics and foot pain in people with knee or hip OA participating in education and supervised exercise. Future investigation should consider the role that foot pain may play on knee and hip related outcomes following therapeutic intervention.

**Supplementary Information:**

The online version contains supplementary material available at 10.1186/s13047-023-00673-5.

## Background and rationale

Osteoarthritis (OA) is the most prevalent chronic joint disease with hip and knee OA causing the greatest burden to the population in terms of pain, stiffness and disability [1]. Due to a lack of effective disease modifying treatments for OA, attention has also turned to identifying modifiable risk factors to help alleviate the disease burden [2, 3]. Understanding the role that clinical characteristics play in knee and hip OA is therefore important and may contribute new insight into the design, conduct or content of interventions to reduce disease burden.

Foot pain is highly prevalent in the general population, with estimates ranging from 13 to 36% [4] and is notably higher in people with radiographic knee OA with estimates of around 25% [5]. Furthermore, the presence of foot/ankle pain in people with symptomatic radiographic knee OA has previously been shown to be associated with increased risk of worsening knee pain over time [6]. Ankle pain with (2.30, 95% CI 1.13 to 4.66) or without (OR: 2.53, 95% CI 1.34 to 4.80) foot pain was associated with > twofold increased odds of incident symptomatic radiographic knee OA and frequent knee pain at up to 84 months follow up [7], indicating the relationship with symptomatic radiographic knee OA may be driven by ankle pain. Other studies have shown that the presence of ankle/foot symptoms in either or both feet increases the risk of developing both incident knee symptoms (1.55, 95% CI 1.10 to 2.19) and incident symptomatic radiographic knee OA (3.28, 95% CI 1.69 to 6.37) over 4 years [8]. Paterson et al. [9] also showed the presence of foot/ankle symptoms was associated with increased risk of knee pain worsening over 4 years in people with symptomatic radiographic knee OA, however no worsening of symptomatic radiographic knee OA was observed.

There is some suggestion of underlying biomechanical mechanisms; where foot pronation causes increased internal tibial rotation and knee adduction moment [10], which in turn may exacerbate existing knee pain [11]. Relationships between pain in multiple joint sites may also be due to factors such as generalised OA [12]. Whilst previous findings suggest there is likely a relationship between foot and knee symptoms, particularly in those with or at risk of knee OA, further exploration would be beneficial. Importantly, little is known about the epidemiology of foot pain in people with symptomatic knee or hip OA, or the potential strength of association between clinical characteristics of this group and foot pain. Thus, the overall aim of this study was to estimate the prevalence of foot pain in people with knee or hip OA and to determine if baseline clinical characteristics were associated with foot pain.

## Methods

### Design, aim and context

Secondary data analysis was conducted on cross-sectional baseline data of individuals enrolled on Good Life with osteoArthritis in Denmark (GLA:D®). GLA:D® was initiated in 2013 with the overarching aim of implementing clinical guidelines for the treatment of knee and hip OA in clinical practice to facilitate high quality care of people with OA in the Danish population. GLA:D® consists of three mandatory elements: a 2-day course for physiotherapists; 8 weeks of patient education and supervised neuromuscular exercise therapy for people with knee or hip OA symptoms delivered by a certified physiotherapist in clinical practice; and data entry into the national GLA:D® registry, with data from baseline, three and 12 month follow-up [13]. Data are objectively measured, therapist-reported and patient-reported [13]. This study, reported according to the STROBE guidelines for reporting cross-sectional studies [8], utilized baseline data to estimate the prevalence of foot pain in people with knee or hip OA participating in GLA:D® and to determine if baseline clinical characteristics were associated with foot pain.

### Ethical approval and consent

According to the ethics committee of the North Denmark Region, ethical approval of GLA:D® was not needed. However, ethics approval for secondary analysis was received from University of Southampton Ethics and Research Online (ethics no: 56534). GLA:D® was approved by the Danish Data Protection Agency (SDU; 10.084). According to the Danish Data Protection Act patient consent for secondary analysis was not required as personal data was processed exclusively for research and statistical purposes.

### Participants and sampling

All participants in the GLA:D® registry have knee and/or hip joint symptoms or functional limitations associated with knee and/or hip OA that have resulted in contact with the health care system. People were enrolled onto the GLA:D® programme based upon having self-reported knee/hip joint problems that resulted in contact with the Danish healthcare system, where they were assessed by the treating therapist to have symptomatic OA [13]. For the current study we included individuals enrolled in GLA:D® between 1st of July 2014 and 8th of April 2018 and who had completed a baseline pain body mannequin. Patients enrolled in GLA:D® after this date were not included as pain mannequin data were not collected due to a database error (9^th^ of April 2018—3^rd^ of November 2020).

### Self-reported, objective clinical, & derived outcome measures

**Fig. 1:**
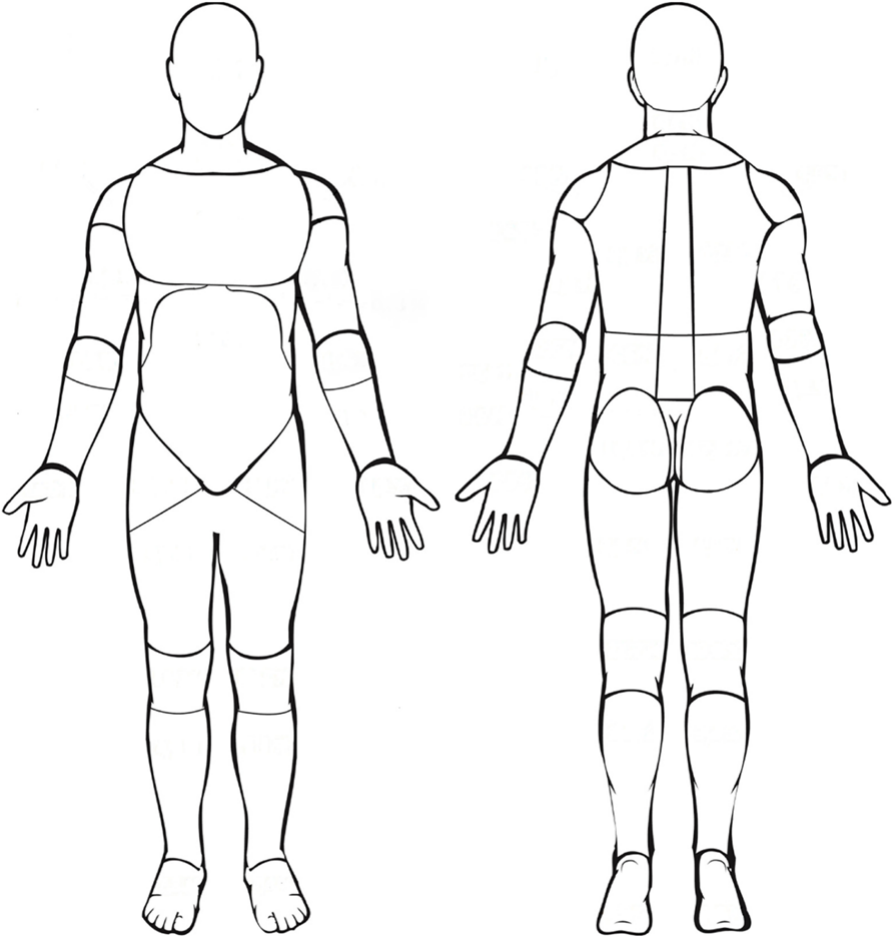
56-site full body pain mannequin. The presence of foot pain was derived from positive indication on a 56-site pain mannequin (4 sites based on plantar or superior aspect of left or right feet)

To explore the association between baseline characteristics and foot pain in those with hip or knee OA, the following variables were selected and stratified by the most affected joint (hip or knee): number of knee/hip joints affected, Knee/hip pain severity (NRS), pain medication use, physical activity level. Knee/hip pain prevalence was also established. These were selected based on prior research based on studies observing correlates with foot pain [14, 15],clinical reasoning and availability within the dataset. Where it was believed other variables may introduce a confounding effect based upon clinical reasoning these were also included where available, these included age, sex and BMI. The presence of foot pain was derived from positive indication on a 56-site pain mannequin (4 sites based on plantar or superior aspect of left or right feet; Fig. 1) indicating where the participant had experienced pain during the last 24 h. The prevalence of foot pain was calculated as a percentage of those with i) pain in either foot, ii) pain in both feet, or iii) pain in neither foot. Index knee and hip pain were derived from specific questions based on the most affected joint (hip or knee) and side (left or right). The prevalence of knee and hip symptoms was based on a positive indication of knee or hip joint being affected and the distribution of knee and hip symptoms based on side affected. The number of painful knee/hip joints were calculated based on a count of 1–4. Pain intensity in the worst knee/hip joint during the past month was captured (VAS 0–100) and use of pain medication in the past three months for knee or hip pain recorded (Yes/No). Current physical activity level was measured using the University of California Los Angeles (UCLA) activity score [16], which assessed activity levels in the past 4 weeks, providing a range of levels from one (wholly inactive) to ten (very high activity). Activity levels were dichotomized as low to moderate activity (UCLA score 1–6) and high activity (UCLA score 7–10) [17]. The presence of depression was derived from the self-reported question “Do you have a depression?” (Yes/No). Sex was derived directly from the Civil Registration number which identifies every citizen in Denmark and age derived from a combination of the Civil Registration number (containing date of birth) and date of initiating the intervention. Body Mass Index (BMI, kg/m2) was calculated from height and weight assessment completed during baseline clinical visit.

### Statistical analysis

Complete case analysis was used due to the low number of missing observations (0.49%) within the main exposure variable (most affected knee/hip joint). Participants who had both outcome and exposure data available and were not missing observations from any potential confounders (age and sex) were included in the analyses. Comparisons were made between those with and without missing data for key variables. Descriptive data for demographic characteristics and baseline foot, knee, and hip pain were calculated using means and standard deviations or frequencies.

Univariable and multivariable logistic regression analysis was undertaken to calculate the strength of association between baseline clinical characteristics (including pain severity in worst knee/hip joint, number of painful knee/hip joints, pain medication use, and physical activity level) and the outcome of foot pain, in those selected for hip or knee OA. Analysis was stratified by those with index knee problems and index hip problems. Directed acyclic graphs (DAG) were used to identify potential confounders and causal relationships between variables, resulting in the exclusion of depression from multivariable models. Multicollinearity was assessed by tolerance and variance inflation factor (VIF). All analyses were carried out in Stata 15.1 (StataCorp, College Station, TX, USA).

## Results

Of 26,003 individuals included in this study, 74.6% (*n* = 19,391) were enrolled with the knee as their most affected joint and 25.4% (*n* = 6,612) with hip as the most affected joint. Of 26,003 included patients (mean age 64.83 ± 9.69 years, mean BMI 28.32 ± 5.26), 72.2% (*n* = 18,784) were registered as female, 4.4% (*n* = 1,137) reported having depression, 63.0% (*n* = 16,370) used pain medication, 64.3% (*n* = 16,706) had a low to moderate ULCA score (see Table 1 for full cohort characteristics).

**Table 1:** Cohort characteristics

**Characteristics**	Complete case (*n* = 26,003)	Index knee Joint (*n* = 19,391)	Index hip Joint (*n* = 6,612)
Age, mean (SD), years	64.8 (9.7)	64.5 (9.7)	65.8 (9.5)
BMI*, mean (SD), kg/m2	28.3 (5.3)	28.7 (5.3)	27.1 (4.8
Sex, F (%) Yes	18,784 (72.2)	13,947 (71.9)	4837 (73.2)
Most affected joint, n (%) Knee	19,391 (74.6)	NA	NA
Pain medication, n (%) Yes	16,370 (63.0)	12,038 (62.1)	4332 (65.5)
Depression, n (%) Yes	1137 (4.4)	867 (4.5)	270 (4.1)
Physical activity level, median (IQR), UCLA*	6 (4, 7)	6 (4, 7)	6 (4, 7)

^*^BMI Body mass index. *UCLA* University of California Los Angeles

Those patients missing data were older (mean difference of 2.5 years) and had lower knee pain intensity scores (mean difference in scores of -5.25). There was a higher percentage of females with missing data (1.2%) than males (0.9%) (see Appendix 1 for missing data table). The overall prevalence of foot pain in people with symptoms of knee or hip OA attending the GLA:D® programme was 12%, this differed slightly according to whether the index joint was knee (12%) or hip (10%).

Irrespective of the index joint (the joint indicated as most problematic on entry into the GLA:D programme), 83.6% (*n* = 21,738) participants reported either left or right knee affected and 38.5% (*n* = 10,017) reported either left or right hip affected. The prevalence of foot pain in those with knee or hip OA was 11.7% (*n* = 3,049). Specifically, 12.4% (*n* = 2,396) of those whose most affected joint was the knee and 9.9% (*n* = 653) of those whose most affected joint was the hip, reported foot pain (see Table 2 for distribution of foot, knee, and hip pain). Of those with left knee symptoms (*n* = 15,328), 11.0% also had left foot pain and 11.5% with right knee symptoms (*n* = 15,535) also had right foot pain, irrespective of index joint. Likewise, in those with left hip symptoms (*n* = 5,825) 10.7% (*n* = 624) also had left foot pain, and of those with right hip symptoms (*n* = 6,386), 11.2% (*n* = 712) also had right foot pain.

**Table 2:** Presence and distribution of foot, knee, and hip pain, stratified by index joint

		Index knee Pain (*n* = 19,391)	Index hip pain (*n* = 6,612)
Foot pain presence, n (%) Yes		2,396 (12.4)	653 (9.9)
Foot pain distribution	Left *	1,947 (10.0)	524 (7.9)
	Right *	1,986 (10.2)	523 (7.9)
	Left (unilateral)	410 (2.1)	130 (2.0)
	Right (unilateral)	449 (2.3)	129 (2.0)
	Bilateral	1537 (7.9)	394 (6.0)
	Either foot	2,396 (12.4)	653 (9.9)
Most affected joint side, n (%) Right		10,104 (52.1)	3,581 (54.2)
Knee/Hip pain severity, mean (SD), VAS		48.12 (22.1)	48.10 (21.5)
Knee pain distribution, n (%)	Left	5,140 (26.5)	1,063 (16.1)
	Right	5,457 (28.1)	953 (14.4)
	Bilateral	8,794 (45.35)	331 (5.01)
	No Knee pain	NA	4,265 (64.50)
Hip pain distribution, n (%)	Left	1,373 (7.08)	2,258 (34.15)
	Right	1,521 (7.84)	2,671 (40.40)
	Bilateral	511 (2.64)	1,683 (25.45)
	No Hip pain	15,986 (82.44)	NA
Number of knee/hip joints affected, n (%)	One	8,080 (41.67)	2,922 (44.19)
	Two	10,284 (53.03)	3,188 (48.22)
	Three	655 (3.38)	333 (5.04)
	Four	372 (1.92)	169 (2.56)

*not exclusive to one side

Adjusted regression analysis showed that the number of painful knee/hip joints and knee/hip pain severity in the worst knee/hip joint, were significantly associated with foot pain. Taking pain medication was also associated with foot pain in those with knee OA (Table 3). Physical activity was negatively associated with foot pain; however, this relationship was attenuated within multivariable analysis.

**Table 3:** Associations between foot pain and baseline characteristics based in adjusted multivariable models

	**Index knee (*n* = 19,391)**						**Index hip (*n* = 6,612)**					
	**Univariable**			Multivariable*			**Univariable**			Multivariable*		
	**OR**	**95% CI**	***p*-value**	**OR**	**95% CI**	***p*-value**	**OR**	**95% CI**	***p*-value**	**OR**	**95% CI**	***p*-value**
Number of painful knee/hip joints	1.76	1.65, 1.87	< 0.001	1.68	1.58, 1.80	< 0.001	1.72	1.55, 1.91	< 0.001	1.64	1.47, 1.82	< 0.001
Knee/hip pain severity in worst knee/hip joint	1.01	1.01, 1.01	< 0.001	1.01	1.00, 1.01	< 0.001	1.01	1.01, 1.02	< 0.001	1.01	1.01, 1.01	< 0.001
Use of pain medication for knee/hip	1.48	1.35, 1.62	< 0.001	1.24	1.13, 1.37	< 0.001	1.31	1.09, 1.56	0.003	1.08	0.90, 1.31	0.397
Physical activity level	0.94	0.92, 0.96	< 0.001	1.00	0.96, 1.01	0.832	0.94	0.90, 0.98	0.007	1.00	0.93, 1.03	0.359

^*^multivariable models adjusted for age, sex and BMI and mutually adjusted for all other exposures

## Discussion

The overall prevalence of foot pain in people with symptoms of knee or hip OA attending the GLA:D® programme was 12%. The number of other painful knee/hip joints and pain severity in worst knee/hip joint, were associated with foot pain for those with knee or hip OA. Use of pain medication was associated with foot pain in those with index knee OA only. No associations were seen for physical activity level and foot pain in either those with index knee or hip pain.

The prevalence of foot pain was slightly lower than previously reported general population prevalence estimates of 13–36% [4]. The definition of foot pain in GLA:D® was based on pain during the last 24h, which does not reflect fluctuations in foot pain over a longer period of time, nor does it consider symptoms such as aching or stiffness unlike other cohort studies [4]. Paterson et al. [5] previously reported a notably higher prevalence of foot pain (25%) than identified in this work, in their cohort analysis of people with symptomatic radiographic knee OA, defined by frequent knee symptoms (including pain, aching, or stiffness in and around the knee on most days of the month for at least one month in the past year) and radiographic evidence of knee OA (Kellgren/Lawrence grade ≥ 2). The difference could be accounted for by variance in sampling strategy between studies, and thus the cohort reported within this study may represent a wider spectrum of disease, including those with potentially earlier pathogenesis and minimal radiographically observable change. This is the first study to determine a prevalence estimate of foot pain in those with symptomatic hip OA and as such similar comparisons cannot yet be drawn.

It is unclear from cross-sectional observation alone whether foot pain precedes, coincides with, or follows the presence of symptomatic knee or hip OA. Clinically, such information could provide useful guidance for future treatment targets and as such future longitudinal studies are recommended. For example, identification of people for whom symptomatic knee OA is likely to precede concomitant foot pain could lead to earlier treatment targeting to minimise foot pain. This study has however highlighted that whilst the direction of this relationship is unclear, the odds of having foot pain is significantly increased with knee or hip pain severity and the presence of pain in more than one hip or knee joint. This highlights the importance of including foot pain measures within the clinical assessment of knee and hip symptoms, particularly in those with a diagnosis of OA. Given that it is known that people with pre-operative foot pain are more likely to have poorer clinically important outcomes following knee arthroplasty [18], the potential for foot pain to negatively impact concurrent knee and hip symptoms and vice versa should therefore be considered.

Participants of the GLA:D® programme with knee or hip OA symptoms and foot pain were more likely to experience worse pain intensity and pain in more sites. Furthermore, those with symptomatic knee OA and foot pain were also more likely to be using analgesic medicine. The findings reported here are consistent with similar prior observations, where worsening knee pain was associated with foot symptoms over four years in those with symptomatic radiographic knee OA [6]. It is noteworthy that factors such as worsening pain have also been associated with poorer outcome or disease progression in previous studies of knee and hip OA interventions [19, 20]. Arguably, based on our findings, future consideration should also be given to the effect that foot health/pain could have upon the likely success of various treatments for knee or hip OA, including exercise and surgical intervention. For example, it is currently unclear to what extent exercise therapy benefits or is limited by foot pain. Future epidemiological studies could seek to build upon the data presented here to explore the wider impact of foot pain upon people living with symptomatic knee or hip OA. We may then consider the need to optimize foot health as a potentially modifiable treatment target for wider determinants of lower limb health or health-related outcomes.

Some of the limitations of this study, such as use of this particular pain question and cross-sectional design, have already been described. In addition, there is a potential lack of generalisability as the current study only includes those who sought education and exercise for their knee/hip OA pain, whilst other factors that were not accounted for (e.g., acute injury to the foot, previous injury or surgery to the foot, footwear, occupation, etc.) may have contributed to the associations found. We do not know the reasons for foot pain, nor the longevity of its presence, and this type of information would be useful within a future longitudinal study to better understand the relationship between foot, knee, and hip symptoms.

## Conclusion

Twelve percent of people with knee or hip OA participating in patient education and exercise reported having foot pain, to which a range of clinical knee and hip characteristics were associated. Foot pain may therefore be an important factor to consider in the assessment of knee and hip OA. Future longitudinal epidemiological research is required to explore the natural history of foot pain in people with OA, and the potential for foot pain to be a modifiable therapeutic target to reduce disease/symptom progression or increase treatment success.

### Supplementary Information

**Additional file 1: Supplementary figure 1.**

## Data Availability

The data that support the findings of this study are available from Søren T. Skou stskou@health.sdu.dk or Ewa M. Roos eroos@health.sdu.dk but restrictions apply to the availability of these data, which were used under license for the current study, and so are not publicly available. Data are however available from the authors upon reasonable request and with permission of Søren T. Skou stskou@health.sdu.dk or Ewa M. Roos eroos@health.sdu.dk on behalf of the GLA:D® programme team.

## Appendix 1. Characteristics of missing versus non-missing participants

**Table:**

**Characteristics**	Complete case (n= 26,003)	Missing values (n=288)	P Value
Age, mean (SD), years	64.8 (9.7)	67.3 (10.9)	0.000*
BMI, mean (SD), kg/m2	28.3 (5.3)	28.2 (5.4)	0.612
Sex, F (%) Yes	18784 (72.2)	223 (77.4)	0.050*
Foot pain presence, n (%) Yes	3049 (11.7)	28 (9.7)	0.293
Knee pain presence, n (%) Yes	21738 (83.6)	129 (79.6)	0.174
Hip pain presence, n (%) Yes	10017 (38.5)	72 (44.4)	0.123
Knee/hip pain intensity, mean (SD), VAS	48.1 (22.0)	42.9 (24.8)	0.001*
Most affected joint, n (%) Knee	19391 (74.6)	117 (73.6)	0.776
Pain medication, n (%) Yes	16370 (63.0)	175 (60.8)	0.444
Depression, n (%) Yes	1137 (4.4)	12 (6.4)	0.180
Physical activity level, median (IQR), UCLA	6 (4, 7)	6 (4, 7)	0.103

t-tests (or Wilcoxon-Mann-Whitney test) for continuous variables and Chi^2^ tests (or Fishers exact) for categorical variable

*Those missing data were older (mean difference of 2.5 years) and had lower knee pain intensity scores (mean difference in scores of -5.26). There was a higher percentage of females with missing data (1.2%) than males (0.9%).
