# Supplementary material for: The prevalence of foot pain and association with baseline characteristics in people participating in education and supervised exercise for knee or hip osteoarthritis: a cross-sectional study of 26,003 participants from the GLA:D® registry
Source: J Foot Ankle Res. 2023 Nov 23;16:83. doi: 10.1186/s13047-023-00673-5 (PMC10666392; doi:10.1186/s13047-023-00673-5)
Supplement: Supplementary file 1 — Additional file 1: Supplementary figure 1. [file 13047_2023_673_MOESM1_ESM.docx]

Foot Pain

Sex

physical activity level

BMI

Depression

pain severity

number of painful knee/hip joints

Pain medication use

Age

Index knee or hip joint

Supplementary Figure 1. The DAG provides visual representation of potential relationships between independent variables (blue line) and dependant variables (green line), effect modifiers (yellow text) and covariates potentially on the causal pathway (red text).
